# Success Rates of Vitrectomy in Treatment of Rhegmatogenous Retinal Detachment

**DOI:** 10.1155/2016/2193518

**Published:** 2016-07-13

**Authors:** Yasser Helmy Mohamed, Kozue Ono, Hirofumi Kinoshita, Masafumi Uematsu, Eiko Tsuiki, Azusa Fujikawa, Takashi Kitaoka

**Affiliations:** ^1^Department of Ophthalmology and Visual Sciences, Graduate School of Biomedical Sciences, Nagasaki University, 1-7-1 Sakamoto, Nagasaki 852-8501, Japan; ^2^Department of Ophthalmology, El-Minia University Hospital, El-Minia 61519, Egypt

## Abstract

*Aim*. To investigate the anatomical success rates of pars plana vitrectomy (PPV) after primary rhegmatogenous retinal detachment (RRD).* Methods*. This retrospective study was conducted between December 2008 and October 2014 at Nagasaki University Hospital. The preoperative data recorded included the lens status, location of the retinal tear, whether a tear was visualized, presence of multiple tears, macula status, presence of peripheral lattice retinal degeneration, and best-corrected visual acuity (BCVA). The primary outcome measures were anatomical (primary and final) and functional success (visual acuity better than 6/60).* Results*. This study evaluated 422 eyes of 411 patients with a mean age of 57.7 ± 11.2 years. The single-operation reattachment rate (primary anatomical success) was 89.8%. The final anatomical success rate was 100% after 2–6 operations (mean = 3.14 ± 1.03). Functional success rate after the primary reattachment operation was 96.7%, while it was 97.2% at the end of the follow-up. Multiple logistic regression analysis of the possible risk factors for the primary anatomical failure showed a significant relation with the 25 G instruments (*P* = 0.002) and the presence of multiple tears (*P* = 0.01).* Conclusion*. The primary anatomical success of PPV for primary uncomplicated RRD was 89.8% and the final anatomical success rate was 100%.

## 1. Introduction

Rhegmatogenous retinal detachment (RRD) is a potentially blinding ocular condition. The prevalence of RRD ranges from 6.3 to 17.9 per 100,000, with the highest incidence found in patients between 60 and 69 years of age [1].

In the early 20th century, Gonin studied the RRD disease process and reported it was the most common cause of retinal detachment [2]. The three prerequisites for the development of RRD include (1) liquefaction of the vitreous, (2) tractional forces that produce a retinal break, and (3) a retinal break through which fluid gains access to the subretinal space. Several well established major risk factors have been described that significantly influence the risk of RRD development, including cataract surgery, high myopia, severe ocular trauma, ocular infections, lattice degeneration, and glaucoma [2].

The aim of retinal reattachment surgery is to seal all retinal breaks via laser photocoagulation or cryotherapy, relieve abnormal vitreoretinal traction, and reattach the neurosensory retina to the retinal pigment epithelium either externally with the application of a scleral buckle (SB) or internally through pneumoretinopexy or pars plana vitrectomy (PPV) and tamponade. High rates of successful anatomical reattachment of the retina have been achieved when using these techniques for treating RRD of medium complexity. However, except in situations where high grade proliferative vitreoretinopathy (PVR), giant retinal tears, posterior retinal breaks, or hazy ocular media preclude successful scleral buckling as the primary procedure, there has yet to be any clear consensus on which of these techniques are optimal [3]. Furthermore, recent advances in the vitrectomy instrumentation and technique have led many retinal surgeons now to prefer the use of PPV for the repair of RRD. Even so, there are certain disadvantages associated with the SB procedure, which include longer operation and anesthesia times, the potential for globe perforation, and the risk of developing postoperative problems, such as diplopia, buckle extrusion, or infection [4].

In 1985, Escoffery et al. were the first to report using PPV without concomitant SB to treat RD [5]. Since then, numerous case series have been published, with the outcomes for a wide variety of patients (single-operation success rate (SOSR) and visual acuity) generally appearing to be comparable to those achieved with SB. Over the past few years, several retrospective series have compared SB, PPV, and/or combined SB/PPV. These series have described a wide variety of clinical situations, with the majority finding no statistically significant difference in the SOSR among the various procedures. In addition, the visual results have also been generally comparable [6].

Overall, the surgeons responsible for developing small gauge vitrectomy or minimally invasive vitreous surgery (MIVS) have continuously strived to minimize the invasiveness without compromising the outcome. Fujii et al. [7] created a complete 25 G transconjunctival vitrectomy system that consisted of microtrocar cannulas and afforded the ease and safety of instrument introduction and withdrawal, as well as an array of integrated 25 G instruments. Fully integrated 23 G and 27 G vitrectomy systems for routine clinical use were first designed by Eckardt [8] in 2005 and then by Oshima et al. [9] in 2010, respectively.

Since 2008, we have used PPV as the treatment of choice for primary RRD in the Department of Ophthalmology at Nagasaki University Hospital and have had a high success rate for the procedure. The aim of our current study was to examine a larger case series and further investigate the anatomical success rates of pars plana vitrectomy (23 G and 25 G PPV) when performed for primary RRD. In addition, this study, which was conducted from December 2008 until October 2014, also attempted to identify prognostic factors for the primary anatomical success rates of the surgical technique.

## 2. Materials and Methods

This retrospective study adhered to the tenets of the Declaration of Helsinki and was conducted in accordance with the Health Insurance Portability and Accountability Act regulations. After identifying subjects diagnosed with primary RRD who subsequently underwent PPV between December 2008 and October 2014 at Nagasaki University Hospital, we then reviewed all of the patient charts. Patients were excluded if their RRD was repaired with SB or pneumatic retinopexy or PPV with encircling band and if they had a follow-up of less than 6 months and had previous vitreoretinal surgery, giant tears, proliferative vitreoretinopathy (PVR) more than grade B, traction retinal detachment, exudative retinal detachment, RD due to penetrating trauma, or RRD in the setting of infectious retinitis.

The main outcome measure was the single-operation reattachment (primary success) rate. In eyes that underwent a single procedure with sulfur hexafluoride (SF_6_) injection, primary success was defined as retinal reattachment at the 6-month follow-up. Primary success rate in eyes treated with silicone oil (SO) injection was defined as a reattached retina at 6 months after the oil removal. Best-corrected visual acuity (BCVA) was recorded and converted to the logarithm of the minimal angle of resolution (logMAR). The preoperative data recorded included the lens status, location of the retinal tear, whether a tear was visualized, multiple tears, macula status, peripheral lattice retinal degeneration, high myopia (refraction of more than −6 diopters or axial length of more than 23 mm), and BCVA. Data obtained during the follow-up visit included the duration of the follow-up, final BCVA, primary success rate, final anatomical success, and whether cataract extraction was performed with PPV. The primary outcome measures were anatomical (primary and final) and functional success. Final anatomical success was defined as retinal reattachment at 6 months after one or more retinal reattachment procedures. Postoperative BCVA was measured (at the 6-month follow-up and at the final visit) and then converted to logMAR units for analysis. Functional success was defined as a postoperative BCVA better than 6/60 (0.1). The rationale for using 6/60 (0.1) was based on both the legal definition of blindness and the difficulty in interpreting functional outcomes solely on the final visual acuity or improvement in acuity, as both could be influenced by the preoperative visual acuity [3]. Data collected from patients who redetached included the number of redetachments and subsequent procedures performed.

### 2.1. Surgical Technique

All patients underwent standard 3-port 23 G or 25 G PPV using a wide angle viewing system, with relief of vitreoretinal traction at the breaks and drainage of the subretinal fluid through existing breaks or retinotomies. Endolaser photocoagulation was applied to completely surround all retinal breaks or retinotomies. In phakic eyes, simultaneous phacoemulsification and intraocular lens (IOL) implantation was a subjective decision made by the surgeon based on clinical judgment and experience with the particular procedure. Any 23 G or 25 G sclerotomy sites that were found to be leaking at the end of the surgery were sutured with a 7-0 Vicryl suture. Intraocular tamponade was achieved with gas (SF_6_ 20%) or with 1,000 centistokes of silicone oil.

### 2.2. Statistical Analysis

Continuous variables were assessed for normality and summarized using the mean (standard deviation) as appropriate. In logistic regression used to estimate the odds ratio (OR) and 95% confidence interval (95% CI) of the primary success in the RRD operation, we used one value as a reference or control arm. Factors that were statistically significant (*P* < 0.05) in the analysis were included in the multivariable models. Statistical analyses were performed using the JMP® version 11 software (SAS Institute Inc., Cary, NC, USA).

## 3. Results

This study evaluated 422 eyes of 411 patients (260 males and 151 females), with a mean age of 57.7 ± 11.2 years (range 19 to 87 years). The mean age of the males was 56.6 ± 11.2 years (range 28 to 87 years), while it was 59.7 ± 10.9 years (range 19 to 87 years) for the females. There was no statistically significant difference between the two groups for the age (*P* = 0.05) (Table 1). The difference in age between the two groups approached statistical significance in which males are younger than females in this study. All patients fulfilled the inclusion criteria and underwent PPV (23 G and 25 G) for primary RRD at Nagasaki University Hospital between December 2008 and October 2014.

The mean patient follow-up was 586 ± 332 days (range 184 to 2110 days). The single-operation reattachment rate (primary anatomical success) was 89.8% (379/422 eyes). Success was defined as the presence of an anatomically flat retina (no subretinal fluid after a minimum 6-month follow-up) without the need for additional procedures. In this study, 43 (10.2%) eyes required additional operations in order to achieve final retinal reattachment. The final anatomical success rate was 100% after 2–6 operations (mean = 3.14 ± 1.03).

**Figure 1:**
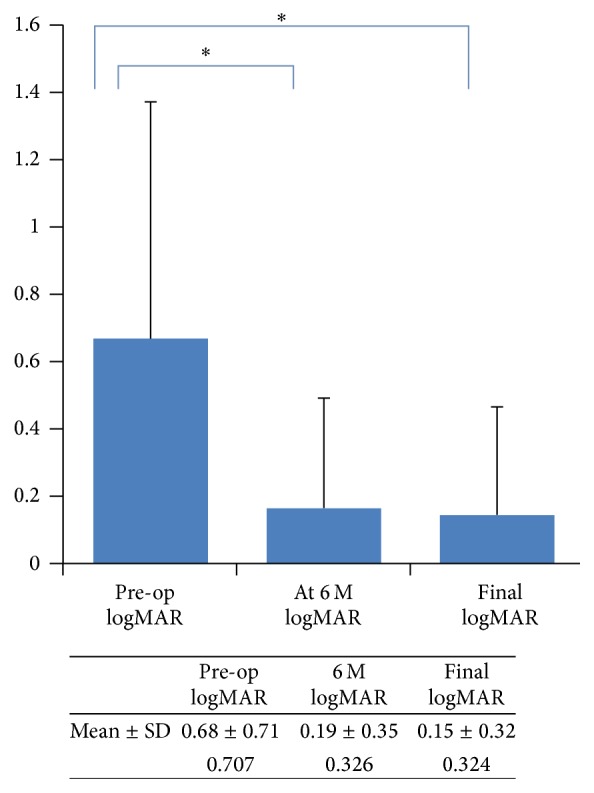
Log MAR of BCVA preop and postoperatively. ^*∗*^Wilcoxon signed rank test *P* < 0.001.

The mean logMAR of the BCVA preoperatively was 0.676 ± 0.7, while it was 0.19 ± 0.3 after the primary retinal reattachment procedure, and 0.15 ± 0.3 after the final anatomical success. Both values were significantly different from the preoperative value (*P* < 0.001 in both cases) (Figure 1).

Functional success rate after the primary reattachment operation was 96.7% (405/419 eyes) (3 eyes were missing visual acuity data at 6 months), while it was 97.2% at the end of the follow-up (410/422 eyes) (Table 2). There was a higher functional success rate compared to the primary success rate, as some of the cases in the failed group had attached macula even though the peripheral retina was still detached.

Logistic regression analysis of the possible risk factors for the primary anatomical failure after one surgical procedure indicated that there was no significant relation for the age and sex of the patients, history of trauma, preoperative lens status (phakia, pseudophakia, and aphakia), high myopia, macula status (on or off), lattice degeneration (presence or absence), preoperative tear visualization, cataract operation during vitrectomy, and location of tear (Table 3).

Primary success rate was 89.8% (316/352 eyes) in the phakic cases and 91.0% (61/67 eyes) in the pseudophakic cases (*P* value = 0.28). Primary success rate was 94.9% (131/138 eyes) in the cases with upper quadrant retinal breaks and 84.0% (21/25 eyes) in the cases with lower quadrant retinal breaks (*P* value = 0.07). The primary success rate was 89.7% (304/339 eyes) in cases of cataract extraction associated with PPV and 90.4% (75/83 eyes) in cases without cataract extraction (*P* value = 0.85).

Univariate logistic regression analysis of the possible risk factors for primary anatomical failure after one surgical procedure showed a significant relation with the 25 G instruments (*P* = 0.04) and the presence of multiple tears (*P* = 0.02) (Table 3).

Multiple logistic regression analysis of the possible risk factors for the primary anatomical failure after one surgical procedure also showed a significant relation with the 25 G instruments (*P* = 0.002) and the presence of multiple tears (*P* = 0.01) (Table 4).

**Figure 2:**
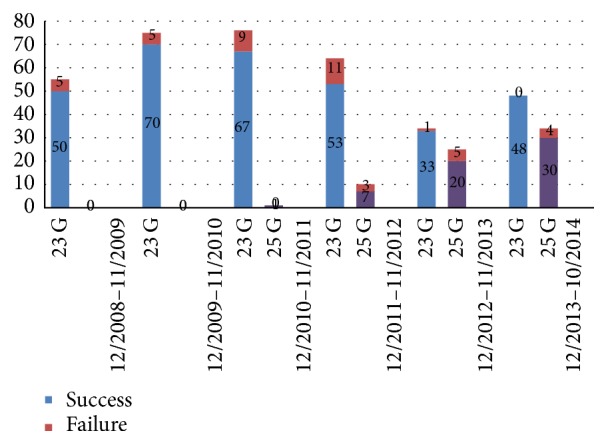
Primary success rate by years of study using PPV (23 and 25 G).

The use of 25 G PPV started during the third year of the current study and gradually increased in frequency especially during the last year of study (Figure 2).

Out of the 352 eyes that underwent 23 G PPV and the 70 eyes that underwent 25 G PPV, the primary success rate was 91.2% in 23 G PPV and 82.9% in the 25 G PPV. The difference between the groups was statistically significant (*P* = 0.04). There was also a statistically significant difference between the two groups with regard to the functional success (*P* = 0.01) (Table 5). Since there was an increased use of 25 G PPV in the treatment of primary RRD over the last three years of the study, we decided to compare the 23 G and 25 G PPV with regard to the primary success rate for this period of time. During the last three years of the study, our analysis showed there was no statistically significant difference between the two groups during this period (*P* = 0.053) (Table 6). When the primary success rate was compared between the 23 G cases during the first three years of the study (90.8%) and the 25 G cases during the last three years (82.6%), we also found that there was no statistically significant difference between the two groups (*P* = 0.07) (Table 7). The difference in success rates between the two groups approaches statistical significance in which the 23 G system is better than 25 G system in treatment of RRD.

## 4. Discussion

If left untreated, RRDis an important cause of visual disability. The precursors of RRD are changes in the vitreous body leading to tractional forces on the retina and the induction of breaks through which fluid can gain access to the subretinal space. To achieve successful retinal reattachment, the goal of surgery for RRD is to treat all retinal breaks and relieve vitreous traction (which may also reduce the incidence of new breaks) [2]. Different surgical techniques have been described to manage this sight threatening condition including pneumatic retinopexy [10], SB, PPV, and a combination of both SB and PPV [11, 12]. For many decades, SB has been the preferred method for surgical repair of uncomplicated primary RRD. Primary PPV was usually reserved for the management of complex retinal detachments. However, PPV has recently been growing in popularity as the preferred surgical procedure for the management of primary uncomplicated RRD [13].

Over the last 40 years, there have been a variety of advancements in vitrectomy surgery including intraocular gases, increased vitreous cutter speeds, wide angle viewing systems, perfluorocarbon liquids, lighted and curved instruments, sutureless vitrectomy, and chandelier lights. These advancements have improved the surgeon's ability to perform a thorough evaluation of the peripheral retina due to the higher magnification and excellent illumination, which is crucial for detecting and treating all retinal breaks and relieving the vitreoretinal traction [14].

Falkner-Radler et al. reported a similar overall trend for vitrectomy in both phakic and pseudophakic RRD, with an increasing use of small gauge vitrectomy for the management of primary RRD [15]. The increasing popularity of vitrectomy can largely be attributed to the advent of transconjunctival sutureless small gauge vitrectomy, which has the advantage of shorter operative times, shorter convalescence, less postoperative inflammation, better patient comfort, and surgical outcome that is comparable with the conventional 20-gauge vitrectomy [3].

Kobashi et al. reported achieving primary anatomical success in 261 (96.3%) of the 271 eyes in the PPV group, with final anatomical success achieved in all eyes [16]. Orlin et al. found that the primary surgical anatomical success rate was 83% in their PPV group and 86% in their PPV/SB group, with all patients (100%) having an attached retina at the most recent follow-up (mean: 406.73 days in the PPV group, 502.14 days in the PPV/SB group) [17]. After Jackson et al. created a National Ophthalmology Database in the United Kingdom (UK) that extracted patient data collected between 2002 and 2010 from participating centers, their subsequent analysis of the data showed that the primary success rate was 87.0% in the PPV group (2693 cases) [18].

A large database study from Taiwan (nationwide population-based study) that analyzed data from 2005 found that the primary success rate after RD surgery was 86.2% [19]. A UK randomized controlled trial (RCT) that examined 615 patients undergoing PPV for RD reported a primary success rate of 84.4% [20]. A population-based Scottish epidemiology study reported a success rate of 81%, excluding cases with silicone oil in situ [21]. Wong et al. examined the primary and final success rates for PPV cases and found rates of 78.6% and 95.2%, respectively [3]. The final anatomical success rates in other reported studies have ranged between 96.6% and 100% [20, 22, 23]. In our current study, we determined the primary and final success rates to be 89.8% (379/422 eyes) and 100%, respectively, with these results comparing favorably to the previously mentioned studies.

In the current study, our analyses indicated that the functional success rate after the primary reattachment operation was 96.7%, which was higher than 72.5% reported by Wong et al. [3]. However, while our study did not contain a control group with PPV, our results were comparable to reports that have been previously published.

When trying to compare study results with previously published reports in the literature, there are potential errors that could be present between the studies, including differences in the baseline patient characteristics, in the surgical technique, and in the experience of the surgeon. Thus, one of the challenges when comparing the success rates found for the different techniques or different populations is determining if the criteria for success vary between the different published reports.

In our study, we determined the primary success rate at 6 months after the primary surgery in cases using SF_6_ and at 6 months after silicone removal in silicone oil cases. Since late redetachments (at 6 months or later following primary surgery) are known to occur infrequently and are often due to vitreous base contraction and new breaks [24]. We think that our 6-month observation point was applicable for our study. Therefore, when redetachments occurred after 6 months, we considered these to be distinct events from the primary surgery, and thus these data were not included in the assessments of the primary success rate. This speculation has been confirmed by Lee et al. who reported that 98.1% (105/107 eyes) of the primary failure cases were diagnosed within 180 days or less [25].

Wong et al. observed that the primary success rates in phakic and pseudophakic groups were 79.0% and 78.2%, respectively, with no statistical difference found between the two groups [3]. Other studies that found the same results for the primary success rate in PPV also reported that there was no relationship between the success and the lens state [16, 17, 26]. In our current analysis, we found the primary success rate was 89.8% (316/352 eyes) in the phakic cases and 91.0% (61/67 eyes) in the pseudophakic cases, with no statistically significant difference found between the two groups. These results contradict those of Caiado et al. who reported finding that the redetachment was significantly higher in the phakic patients than the pseudophakic patients [27]. However, it should be noted that the study by Caiado et al. only included a small number of patients (96 cases). Caiado et al. acknowledged that the number of cases in their study was a potential limitation and they require further studies in order to confirm their findings.

Kobashi et al. [16] reported that although eyes with superior breaks exhibited slightly better results than eyes with inferior breaks, the primary anatomical success rate of the repair of RRD did not significantly differ between the eyes with inferior and superior breaks, which was in line with other previous studies [14, 17, 28]. On the other hand, Goto et al. reported that the anatomical success rate of primary vitrectomy for RRD with inferior breaks was lower than that of RRD with superior breaks (80% versus 98%) [29].

In our study, we assured that the primary success rate was 94.9% (131/138 eyes) in cases with upper quadrant retinal breaks and 84.0% (21/25 eyes) in cases with lower quadrant retinal breaks. Analysis showed no statistically significant difference between the two groups. Several studies have reported finding that PPV combined with cataract surgery offered significant advantages to both patients and surgeons with regard to the management of the RRD [27, 30, 31]. Although we believe that phacovitrectomy and intraocular lens implantation is effective and can help avoid the difficulties that occur after vitrectomy cataract surgery, our study did show there were no statistically significant differences for the primary anatomical success rates between phacovitrectomy and PPV alone (*P* = 0.85).

Dell'Omo et al. performed a logistic regression analysis of the possible risk factors for anatomical failure after a single surgical procedure and reported that there was no significant association for the age of the patients, total number of breaks/holes, number of inferior breaks/holes, preoperative lens status, or gauge of the instruments used for the PPV (20 and 25 G) [26].

With the exception of the association between the multiple breaks and the gauge of the instruments used for the PPV, our study results were in agreement with the study performed by Dell'Omo et al. [26]. Our results did confirm that multiple breaks were considered to be a risk factor of primary failure by both the univariate (*P* = 0.02) and multivariate (*P* = 0.01) logistic regression analysis. We also observed that the use of 25 G PPV had a bad prognostic value as compared to the 23 G PPV for both the univariate (*P* = 0.04) and the multivariate (*P* = 0.002) logistic regression analyses. Furthermore, we also showed that there was a statistically significant difference between the 23 G and the 25 G with regard to both the primary anatomical success (*P* = 0.04) and the functional success (*P* = 0.01) rates. We believe that the statistical difference between these two groups during the 6-year study period was related to the large number of cases for the 23 G PPV (352 eyes) versus the 25 G cases (70 eyes). Although there was an increase in the use of 25 G PPV in the treatment of primary RRD during the last three years of our study, our comparisons of the two systems during just the last three years of the study found no statistical difference between the two systems (*P* = 0.053). To determine if the difference between the 23 G and 25 G systems could be related to the experience of the surgeon performing the technique, we compared the primary success rate in the 23 G cases during the first three years of the study (90.8%) with the 25 G cases during the last three years (82.6%). However, our findings indicated that there was no statistically significant difference between the two groups (*P* = 0.07). The difference between the two groups approached statistical significance which confirms that success rate with 23 G system is better than 25 G system. In addition, the experience of the surgeon in using 23 G system is better than that of 25 G system. To the best of our knowledge, there are no other studies in the literature that have compared 23 G PPV and 25 G PPV. Thus, we were not able to compare our results with any other reports. In the few studies that have compared 20G with the small gauge system (23 G and 25 G), there have been no significant differences found for the primary and final success rates [17, 26]. A further prospective randomized study will need to be undertaken in order to clarify the prognostic factors for the primary anatomical success rates for both gauges (23 G and 25 G) and the preoperative characteristics.

Other studies have determined that neither the macula state nor the lattice degeneration can be considered as risk factors for the primary success rate in RRD surgery [14, 17]. Our current results are in agreement with these studies. Moreover, our study also demonstrated that the age, sex, history of trauma, high myopia, and tear visualization were not risk factors for the primary success in PPV for primary RRD.

Although scleral buckle surgery was once the most commonly performed RD surgery, the most predominate surgery in use today is PPV. For example, approximately three-quarters of the RDs in the United States have been treated by PPV [3]. In the Retina 1 Project, De La Rúa et al. examined the variations in the management of primary RRD in Spain from 1999 to 2006 and found an increasing tendency for the use of PPV as the primary treatment, which was independent of both the clinical characteristics and the surgical outcome [32]. Between 2005 and 2011, it also has been reported that there was an increasing trend for using PPV and PPV + SB as the primary retinal reattachment surgery [3]. Recent meta-analysis studies that examined the management of primary RRD have demonstrated that PPV achieved more favorable effects with less intra- or postoperative complications than SB [6, 18, 33].

In 2002, Fujii et al. developed transconjunctival sutureless vitrectomy (TSV) using a 25-gauge incision, which has turned out to be one of the most innovative vitreoretinal surgical techniques introduced in recent years [7]. In this procedure, three polyamide microcannulas are inserted transconjunctivally through the sclera in the area of the pars plana. Vitreoretinal instruments and infusion lines are then introduced through these cannulas into the vitreous cavity. Because thin 25-gauge instrumentarium is used during this procedure, the incisions left in the sclera after removal of the cannulas are so small that seal without the need for suturing. As a result, this minimizes surgically induced trauma and decreases the convalescence period, operating time, and postoperative inflammatory response [7]. However, current evidence supports the use of 25-gauge TSV only in less complicated vitreoretinal surgery. Indeed, one of the most frequent objections for the use of TSV is that the 25-gauge instruments are too flexible for many of the complicated tasks that need to be performed on the retina and vitreous body, including RRD. Thus, the introduction of a 23-gauge system could help overcome this flaw [8]. These previous findings and speculations may help to explain the superior results we found when using the 23 G PPV versus the 25 G PPV in RRD.

Up until a few years ago, vitreoretinal surgeons in our department preferred to use 23 G PPV for the treatment of RRD. This system was the method of choice due to the easy implantation of the different instruments through the wider pore sclera cannulas and the ability to achieve good shaving of the vitreous when using a 23 G cutter. Today most surgeons have now switched to 25 G PPV system for the treatment of RRD. As seen in Figure 2, this transformation can be undertaken, which demonstrates why there was a gradual increase in the number of 25 G PPVs performed during the last three years of our study. In our opinion, this trend will continue to increase until this system becomes the primary treatment of choice for RRD.

One of the obvious factors that encourage the use of PPV is attributable to the high speed vitrectomy system that is used to carry out all of the surgical procedures. Both the cut velocity along with the cutter gauge and vacuum rate are known to be important factors in determining how much traction is created on the retina during the vitrectomy. The use of a high frequency cutting rate may be critical in helping to avoid iatrogenic retinal breaks that are predisposed to redetachment. Recent advances in instrumentation, surgical adjuvants, and techniques have led to the improvement of the final anatomical success rate of RRD repair so that it is now more than 90%. In addition, the ability to be able to use a chandelier light pipe in a fourth sclerotomy makes it possible for the surgeon to perform self-indentation of the sclera and improve the identification and removal of the vitreous base. This is called “near complete vitreous removal” (NCVR) and is a fundamental step for ensuring the success of PPV in the management of RRD [27].

There were some limitations for our current study. First, this was a retrospective study and second there was no control group that could be used to compare the PPV with our currents results and those in the previously published literature. In addition, because of the inherent differences in patient characteristics, follow-up periods, and outcome definitions, this makes it difficult to compare our outcomes with other studies. A further prospective randomized study will need to be performed in order to clarify additional prognostic factors for the primary anatomical success rates in PPV for different gauge systems and preoperative characteristics. In conclusion, PPV has been growing in popularity as the preferred surgical procedure for the management of primary uncomplicated RRD.

## Figures and Tables

**Table 1:** Demographics of the patients in the study.

	Male	Female	Total	*P* value
Number of cases	260	151	411	
Ages	56.6 ± 11.2	59.7 ± 10.9	57.7 ± 11.2	0.05^*∗∗*^
Mean follow-up period	566 ± 321	599 ± 337	586 ± 332	0.33^*∗*^

^*∗*^Mann-Whitney *U* test &  ^*∗∗*^Student's *t*-test.

**Table 2:** Functional success after 6 months and at final follow-up.

BCVA (decimal)	Yes	No	Total
Improved ≧0.1 (6 months)	405/419 (96.7%)	14/419 (3.3%)	419^#^
Improved ≧0.1 (final)	410/422 (97.2%)	12/422 (2.8%)	422

^#^3 cases lost data of VA after 6 M of follow-up. BCVA in decimal measurement.

**Table 3:** Univariate logistic regression analysis of possible risk factors for primary anatomical failure.

		Reattached	Detached	OR	95% CI	*P* value
		*N* = 379	*N* = 43			
Sex	F	139 (36.7%)	14 (32.6%)	Ref		
	M	240 (63.3%)	29 (67.4%)	1.20	0.62–2.41	0.59

Age	<40	21 (5.54%)	2 (4.65%)	Ref		
	40–49	58 (15.3%)	8 (18.6%)	1.45	0.33–10.1	0.64
	50–59	129 (34.0%)	14 (32.6%)	1.14	0.29–7.59	0.86
	60–69	121 (31.9%)	14 (32.6%)	1.21	0.31–8.09	0.80
	≧70	50 (13.2%)	5 (11.6%)	1.05	0.21–7.73	0.96

Lens status	Aphakia	2 (0.5%)	1 (2.3%)	Ref		
	IOL	61 (16.1%)	6 (14.0%)	0.20	0.02–4.59	0.26
	Phakia	316 (83.4%)	36 (83.7%)	0.23	0.02–4.96	0.28

History trauma	(−)	365 (96.3%)	41 (95.4%)	Ref		
	(+)	14 (3.7%)	2 (4.6%)	1.27	0.20–4.77	0.76

High myopia	(−)	244 (64.4%)	28 (65.1%)	Ref		
	(+)	135 (35.6%)	15 (34.9%)	0.97	0.49–1.85	0.92

Macula status	On	187 (49.3%)	18 (41.8%)	Ref		
	Off	192 (50.6%)	25 (58.1%)	1.35	0.71–2.59	0.35

Lattice	(−)	171 (45.1%)	23 (53.5%)	Ref		
	(+)	208 (54.9%)	20 (46.5%)	0.71	0.37–1.35	0.30

Tear presence	Yes	334 (88.1%)	38 (88.4%)	Ref		
	No	45 (11.9%)	5 (11.6%)	0.97	0.32–2.40	1.02

Cataract operation	(−)	75 (19.8%)	8 (18.6%)	Ref		
	(+)	304 (80.2%)	35 (81.4%)	1.08	0.50–2.59	0.85

Tear status	Upper	131 (34.8%)	7 (16.3%)	Ref		
	Temporal	64 (17.0%)	8 (18.6%)	2.34	0.80–6.95	0.12
	Nasal	11 (2.9%)	2 (4.7%)	3.40	0.47–16.3	0.20
	Lower	21 (5.6%)	4 (9.3%)	3.56	0.87–12.9	0.07
	Multiple	149 (39.6%)	22 (51.2%)	2.76	1.20–7.12	0.02^*∗*^

PPV system	23 G	321 (84.7%)	31 (72.1%)	Ref		
	25 G	58 (15.3%)	12 (27.9%)	2.14	1.00–4.32	0.04^*∗*^

^*∗*^Statistically significant difference.

**Table 4:** Multiple logistic regression analysis of possible risk factors for primary anatomical failure.

		Odds ratio	95% CI	*P* value
PPV system	25 G	4.53	1.67 to 13.0	0.002^*∗*^
	23 G	Reference		

Tears status	Multiple tears	2.95	1.25 to 7.85	0.01^*∗*^
	Upper	Reference		
	Temporal	2.60	0.87 to 8.03	0.08
	Nasal	4.70	0.62 to 24.3	0.124
	Lower	3.50	0.80 to 13.7	0.09

^*∗*^Statistically significant difference.

**Table 5:** Comparison between 23 and 25 G along the whole study period.

	23 G	25 G	Total	*P*
Primary anatomical success	321/352 (91.2%)	58/70 (82.9%)	379/422 (89.8%)	0.04^*∗*^
Functional success	345/352 (98.0%)	64/70 (91.4%)	411/422 (97.4%)	0.01^*∗*^
Final success	352/352 (100%)	70/70 (100%)	422/422 (100%)	

^*∗*^Fisher's exact test.

**Table 6:** Comparison between 23 and 25 G in last 3 years of the study.

Last 3 years	Reattached	Detached	OR	95% CI	*P*
23 G	134/191 (70.2%)	12/24 (50.0%)	Ref.		
25 G	57/191 (29.8%)	12/24 (50.0%)	2.35	0.99–5.60	0.053

**Table 7:** Comparison between 23 G group (first 3 years) and 25 G group (last 3 years).

	Attached	Detached	Unadjusted OR	95% CI	*P*
23 G (first 3 years)	187/206 (90.8%)	19/206 (9.2%)			Ref.
25 G (last 3 years)	57/69 (82.6%)	12/69 (17.4%)	2.07	0.92–4.48	0.07
